# Determinants of Unsafe Plastic Waste Disposal among Households in the Tamale Metropolitan Area, Ghana

**DOI:** 10.1155/2021/9974029

**Published:** 2021-10-07

**Authors:** Emmanuel Kombiok, Kingsley Atta Nyamekye, Rita Adjei, Leslie Danquah

**Affiliations:** ^1^Environmental Planning and Development Programme, Department of Planning and Sustainability, School of Geosciences, University of Energy and Natural Resources, Sunyani, Ghana; ^2^Department of Planning and Sustainability, School of Geosciences, University of Energy and Natural Resources, Sunyani, Ghana; ^3^Department of Geographical Science, School of Geosciences, University of Energy and Natural Resources, Sunyani, Ghana

## Abstract

**Background:**

The global discourse on plastic waste generation and disposal has over the last two decades, gained traction with the aid of research-based evidence. Though observed globally, the situation is quickly deteriorating in developing countries such as Ghana. In Ghana and Africa as a whole, rapidly increasing population and rural to urban migration have been cited as factors that exacerbate the existing struggles with plastic pollution. This study aimed at identifying the determinants of unsafe plastic waste disposal among households.

**Methods:**

The study was carried out in three communities in Tamale in the Northern Region of Ghana. Data were collected from 270 randomly selected households through household surveys, key informant interviews, and direct field observations.

**Results:**

The study revealed that the majority (63.3%) of the total respondents used and disposed of their plastic waste “unsafely.” The analysis showed that the education level and household wealth were significant determinants of unsafe plastic disposal.

**Conclusion:**

The study concludes that challenges of plastic waste management are not limited to economic, technical, and institutional factors, but social factors such as human behavior are key aspects of waste management that need attention. The study, therefore, recommends strict enforcement of sanitation by-laws, promotion of education, and provision of alternatives to plastics that will minimize the need for importing and manufacturing plastics, as potential steps towards addressing unsafe disposal of plastics in the domestic environment.

## 1. Introduction

Solid waste disposal, a global challenge, has manifested exponentially in developing countries such as Ghana. This is a result of a combination of various factors that influence how solid waste, specifically, plastics from households is handled. Despite the numerous strategies for the disposal that have been proffered around the world, the uniqueness of various countries and their people makes it impossible for the same proposed methods of disposal to work effectively and efficiently under varying conditions. Unsafe plastic waste disposal in both developed and developing countries are characterized by many factors that are economic, sociodemographic, and environmental [1]. The rapid increase in urban populations has been largely attributed to rural-urban migration. This has an effect, contributed to the increase in waste generated in these urban areas, subsequently affecting the adequate collection and safe disposal of plastic and other forms of solid waste [2]. Also, the bad road networks compound the already glaring challenges of waste management service provider's ability to get to these areas to collect waste; this situation is worse off during the rainy season, and therefore, residents opt for unsafe ways of disposing of their waste when the waste bins are full [3]. The situation in low-income suburban areas is worse because of the already existing inappropriate waste disposal methods. The attention and efforts by local authorities tend to concentrate on central business districts and the more affluent communities that have better access. Improving economic conditions especially in developing countries gives way to increasing amounts of waste generated, and therefore, generation of waste and safely disposing of it is highly influenced by the income levels of households in terms of affording waste management of services [4, 5].

It has been observed in the Tamale Metropolitan Area that communities inhabited by low-income earners are being gradually converted into dumping sites for all categories of waste, including plastic waste. Meanwhile, dumping sites situated at safe distances outside municipal borders are slowly being encroachments by settlements [6]. The locations of natural resources such as rivers, streams, and other features such as topology and drainage of land areas can influence the waste disposal decisions of people. Studies have captured the deliberate disposal of plastic waste into rivers and streams with the anticipation of it being washed away [7]. Behind the disguise of reclaiming an area degraded by erosion, some residents resort to dumping waste into gullies created by erosion, and in some cases, plastic bags are used as sandbags to block waterways resulting from surface runoffs. Due to the resilience of plastics, ecosystems in environments such as oceans and rivers can be harmed, and this has pushed for studies in efforts to understand the issue [7]. Jambeck [8] reported that an amount ranging between 1.1 and 8.8 million metric tons of plastic is introduced into the ocean annually from land sources from the leading 20 countries that mismanage 83% its total waste. According to Ellsbury [9], reusing plastic bottles designed to be used once can increase the probability of BPA leaching, and hence, highly risky. Hence, in trying to reduce the consumption of single-use plastic bottles safely, reusable bottles appear to be a viable option.

The management of solid waste is a challenge in most developing countries when compared to developed countries with the distinction not solely on the composition of waste but also on the availability and quality of the services being provided [10]. The focus of developing countries with regards to waste management is achieving proper collection and disposal, whereas the developed countries are transforming waste into resources as posited [11–13]. The use of landfills becomes the last resort when waste has not been prevented, diverted, or recovered in the previous steps [14, 15]. The new paradigm in waste management is focused on compliance with waste management regulations. It emphasizes on improving waste management in the form of preventing waste generation as well as exploiting waste as a resource [16–18]. Commercially available reusable bottles are available in different materials such as plastic, stainless steel, aluminum, and glass with each possessing its own advantages and disadvantages over the others; however, irrespective of the material component, they all contribute to the reduction of single-use plastics [19–21].

The menace of plastic pollution in the Tamale Metropolitan Area is characterized by indiscriminate dumping, irregular collection of waste, and inadequate resources to ensure efficient service from service providers. This study sought to highlight the determinants of unsafe plastic disposal methods within individual households to determine its relationship with the current sanitation issues plaguing the study area and the nation as a whole.

## 2. Materials and Methods

### 2.1. Study Area

This study was carried out in three selected communities in the Tamale Metropolitan Area of the Northern Region of Ghana. The Buipela and Kanvili communities are located within the Tamale Metropolis, whereas Fuo is located within the Sagnarigu District. As per the 2010 housing and population census, the three communities had combined about 1,040 households [22, 23].

### 2.2. Sampling Procedure

The estimation of the sample size was achieved from a sample frame of 1,040 households from which 270 households were selected using the mathematical formulae for determining the sample size [24]. The sample size was then proportionally distributed to obtain the number of households to be surveyed within the three selected communities. Consequently, 109, 132, and 29 households were selected in Builpela, Kanvili, and Fuo, respectively. Using aerial photographs of the communities, each housing unit in each community was numbered serially. Simple random sampling was then employed to select the number of housing units corresponding to the number of households required for each community with the assumption that each housing unit contained at least a household.

### 2.3. Data Collection and Analysis

Semistructured questionnaires were administered to households to investigate the strategies adopted by households to manage the plastic waste generated within the households. The key areas of the questionnaires were age, household size, educational level, household wealth, types of plastic waste generated daily, the volume of plastics per day, and availability of disposal facilities and services. These data were collected in order to provide critical empirical evidence of determinants of plastic waste pollution as captured by other studies. These data are necessary in achieving the objectives of the study. Statistical analyses were performed using chi-square and logistic regression to determine the relationship and predictability of the dependent variable by the independent variables as reported in Tables 1 and 2, respectively. To facilitate the analysis process, a desk study approach was used to review relevant literature for the study.

## 3. Results and Discussion

The findings of the study covered the derivation of safe and unsafe plastic waste disposal methods employed by the respondents relying on the suggestions of Wilson et al. [25]. It also highlights the relationship between the sociodemographic characteristics of the respondents and other relevant factors and disposal of plastic waste. The findings further established statistically the determinants of unsafe plastic waste disposal by the respondents in the study area.

### 3.1. Unsafe Plastic Waste Disposal

The Global Waste Management Outlook by Wilson et al. [25] for the United Nations Environment Programme provided some background on the strategies for combating waste management issues. With information on waste collection diversity and relevant strategies as a background, any method of disposal (burning and open dumping) that excluded “Pick up by garbage truck” were categorized as “unsafe.” From the findings of the study, it was observed that 171 (63.3%) of the total respondents used and disposed of their plastic waste “unsafely,” whereas 99 (36.7%) disposed of their plastic waste “safely.” The categorization indicated above was derived from the respondent's choices of disposal methods as given in Table 3.

The chi-square analysis highlighted the relationship between unsafe plastic waste disposal and sociodemographic characteristics of the respondents, as well as other external factors as suggested by other researchers that numerous factors that contribute to unsafe disposal of waste. Additional factors that were considered included types of plastic waste, the volume of plastics in total waste generated daily, the availability of waste disposal facilities, the availability and use of garbage collection services, knowledge, attitude, practices of respondents, and the conscious efforts to minimize plastic use.

From the findings, Table 1 provides the statistically significant (*p* ≤ 0.001) relationship between the educational level and unsafe plastic waste disposal at *p* < 0.05. It is also observed that employment status has a significant (*p* ≤ 0.040) relationship with unsafe plastic waste disposal.

### 3.2. Analysis of Determinants of Unsafe Plastic Waste Disposal

The findings in Table 2 show that the educational level and socioeconomic status of respondents are statistically significant determinants of unsafe plastic waste disposal at *p* ≤ 0.001 and *p* ≤ 0.003, respectively. However, age of respondents, the volume of plastic waste generated, types of plastic waste, household size, and availability of plastic waste disposal facilities are not significant determinants of unsafe plastic waste disposal.

### 3.3. Discussion

#### 3.3.1. Determinants of Unsafe Plastic Waste Disposal: An Interplay of Various Factors

The importance of education cannot be oversimplified when discussing issues of waste disposal [10]. The knowledge and understanding of the risk and health implications resulting from improper disposal of waste is a major factor in shaping the mindset of a person. This therefore underscores the importance of a person's level of education generally choosing safe disposal methods for the waste they generate. Contrary to this assertion, the findings of this study showed that 53.3% of the respondents with a minimum of SHS level in education disposed of their plastic waste using unsafe methods. It is therefore imperative to note that the level of education attained by a person does not necessarily translate into safer disposal options, as it also requires discipline and a positive attitude towards a clean environment. They have observed instances where people who had little or no education at all demonstrated the need to dispose of waste safely by traveling a kilometer or more by foot to access communal containers that serve as a waste collection point by local authorities. Notwithstanding the contradicting findings by various studies regarding the role of education in waste management, this study posits that there is the need to incorporate and or intensify educational programs and courses to sensitize people, both formally and informally at every level of education.

As established as a significant determinant of unsafe plastic waste disposal in this study, household wealth plays an important role in the choice of disposal methods by households. Income levels of households are the key because safe disposal methods are mostly charged or levied. Therefore, people with employment and steady income sources can afford private bins and collection services from both state and private waste service providers. Afroz and Masud [4] posits “High-income households generate more waste that can be attributed to high consumption rates among the high-income areas than low-income areas.” This study agrees with this assertion while suggesting that the availability of resources enables the adoption of safer disposal methods as not the case in lower-income areas as revealed in this study. Waste management companies are mostly engaged by high-income households to provide waste bins in the houses of their clients and subsequently go to collect the waste with their trucks promptly. This approach is effective and efficient for waste collection and disposal because the service providers are accountable to their clients and may lose them due to other companies should avoid their clients be unsatisfied with their performance.

The location of households is a factor that is being overlooked. The areas of low-economic activities, largely occupied by low-income earners are gradually being converted to waste dumping sites for plastic waste and more. Similarly, our findings show and affirm that dumping sites located at safe distances from municipal boundaries are being gradually swallowed up by settlements [26, 27]. Besides, natural features such as topology and drainage of the land area can influence resident's choice of waste disposal methods. This study found that people intentionally dispose of plastic waste into rivers and streams in anticipation of it being washed away by the currents. Some people also, in the disguise of reclaiming an eroded area, resort to dumping all sorts of waste into the gullies created during erosion. In some cases, plastics are used as sandbags to block and redirect new waterways created from surface runoffs.

#### 3.3.2. The Evolving Challenges of Waste Disposal

Developing countries have been diagnosed with severe challenges in waste management; hence, it is imperative to examine the numerous factors that constrains waste management service providers and consequently frustrates their attempts to manage waste efficiently. Puopiel and Owusu-Ansah [10] and Zurbrugg [28] have established extensively that the challenges plaguing waste management in developing countries include but are not limited to financial, technical, and institutional issues. The current trends and observations in this study go along to buttress this assertion, further highlighting the need for an integrated approach in tackling waste management issues in Ghana.

The study finds that solid waste management planning and operation is characterized by inadequate human resources at both the national and local levels, coupled with the insufficient technical expertise necessary for success. Many of the officers tasked with waste management at the local levels possess little or no technical knowledge and expertise to effectively perform. This revelation, therefore, emphasizes the need to educate and train human resources to make meaningful contributions to alleviate the challenges of waste management.

It is critical to note that solid waste management, especially plastics, despite the overwhelming evidence, has not been given the high priority it deserves in developing countries except maybe in capital or biggest cities of strategic interest. Therefore, not enough funds are made available to the solid waste management sector by the governments, and this reflects poorly in the levels of service delivery necessary for the protection of the environment and promotion of public health. This problem is severe at the local government level as it is exacerbated by an inadequately developed taxation system resulting in a weak financial basis for public services, especially solid waste management. However, user service charges can cushion the financial shortcomings if the ability of users to procure these services is not restricted and their willingness to procure the said services, more regular and effective [28, 29].

Notwithstanding the involvement of several agencies at the national level regarding waste management, no benefits are being realized due to the unclear definition of roles and functions of the participating agencies. Also, no single agency or committee is assigned coordination duties of projects and activities, and this may be attributed to the fragmentation of legislation on solid waste management [15, 30].

In the developing world, a smaller portion of the urban population has access to dependable waste collection schemes. Periurban dwellers of low-income status mostly do not have access to waste collection or management services, and a key reason is the lack of financial means. People cannot afford the already inadequate fees charged, and the authorities do not have enough funds to ensure that waste management services are available to all. Discounting financial constraints, there is sufficient evidence that operational inefficiencies of waste services providers such as deficient management capacity of the institutions and inappropriate technologies affect effective waste management. Therefore, it is critical that a developing country like Ghana underscores the key challenges of waste management, which include financial and institutional constraints to adequately provide systems to tackle the waste menace.

## 4. Conclusion

This study discussed the unsafe disposal of plastic waste in the Tamale Metropolitan Area amidst a continuous increase in urbanization. The study has revealed that the educational level and employment status of respondents have positively significant relationships with unsafe plastic waste disposal. This study established that education could not solely mitigate the unsafe disposal of plastic waste; however, the combination of factors such as employment, socioeconomic status, and knowledge will have a positive influence on the unsafe disposal of plastics. The study also concludes that the education level and household wealth are statistically significant determinants of unsafe plastic waste disposal. However, an increase in the education level of respondents does not necessarily translate into safe plastic waste disposal as many factors come into play as already determined in discussion. In response to the observed multifaceted challenges of plastic waste disposal in the Tamale Metropolitan Area, policymakers in local governments and other relevant institutions must prioritize improving the knowledge, attitude, and practices of people towards plastic waste management as this study highlights that education is a determinant of unsafe plastic waste disposal. In the long term, policies must capture exploring the possibilities of making plastic waste gathering an informal source of income on a commercial scale. This will improve the environment while providing jobs for many young unemployed Ghanaians, therefore, creating a vibrant “plastic waste market” that will birth benefit towards improving the environment and the socioeconomic standards of the people, hence, improving household wealth, which is a determinant of unsafe plastic waste disposal. Future research studies should prioritize alternatives to plastics that can bring some relief to the environment and human health in general.

## Figures and Tables

**Table 1:** Analysis of unsafe plastic waste disposal.

	Disposal of plastic waste		*X* ^2^ statistic	*P* value (*P*≤)
Age of the respondents	Safe	Unsafe		
Below 35	22 (33.8%)	43 (66.2%)	0.293	0.588
35 and above	77 (37.6%)	128 (62.4%)		

Household size				
3 and below	2 (22.2%)	7 (77.8%)	0.836	0.360
Above 3	97 (37.2%)	164 (62.8%)		

Educational level				
JHS and below	8 (10.7%)	67 (89.3%)	30.230	0.001^*∗∗∗*^
SHS and above	91 (46.7%)	104 (53.3%)		

Household wealth				
High	35 (43.2%)	46 (56.8%)	2.249	0.325
Middle	36 (35.0%)	67 (65.0%)		
Low	28 (32.6%)	58 (67.4%)		

Types of plastic waste generated daily				
Easily degraded	45 (41.7%)	63 (58.3%)	1.938	0.164
Not easily degraded	54 (33.3%)	108 (66.7%)		

The volume of plastics in total waste per day				
Less than 60%	46 (38.3%)	74 (61.7%)	0.258	0.611
60% and above	53 (35.3%)	97 (64.7%)		

Availability of waste disposal facilities				
Yes	6 (37.5%)	10 (62.5%)	0.005	0.943
No	93 (36.6%)	161 (63.4%)		

Usage of garbage collection services				
Yes	77 (36.3%)	135 (63.7%)	0.009	0.925
No	13 (37.1%)	22 (62.9%)		

Minimization of plastic use				
Yes	17 (40.5%)	25 (59.5%)	0.311	0.577
No	82 (36.0%)	146 (64.0%)		

Knowledge				
High	42 (39.3%)	65 (60.7%)	5.532	0.063
Moderate	51 (39.2%)	79 (60.8%)		
Low	6 (18.2%)	27 (81.8%)		

Attitude				
Positive	11 (44.0%)	14 (56.0%)	1.715	0.424
Neutral	6 (50.0%)	6 (50.0)		
Negative	82 (35.2%)	151 (64.8%)		

Practices				
Good	13 (32.5%)	27 (67.5%)	0.579	0.749
Moderate	41 (39.0%)	64 (61.0%)		
Poor	45 (36.0%)	80 (64.0%)		

Employment				
Yes	25 (28.2%)	64 (71.9%)		0.040^*∗*^
No	74 (40.9%)	107 (59.1%)	4.206	

^∗^Significance level *α* = 0.05.

**Table 2:** Regression of determinants of unsafe plastic waste disposal.

Determinants	B	S.E.	Wald	Df	Sig.	Exp. (B)
Age of respondent	0.152	0.332	0.211	1	0.646	1.165
Education level	2.342	0.423	30.708	1	0.001^*∗∗∗*^	10.398
Household size	0.628	0.902	0.485	1	0.486	1.875
The volume of plastic waste generated per day	−0.318	0.281	1.276	1	0.259	0.728
Types of plastic waste generated daily	−0.456	0.283	2.600	1	0.107	0.634
Availability of plastic waste disposal facilities	−0.237	0.600	0.156	1	0.693	0.789
Household wealth	−0.893	0.304	8.629	1	0.003^*∗∗*^	0.410

^∗∗^Significance level *α* = 0.05. ^∗∗∗^Significance level *α* = 0.01.

**Table 3:** Plastic waste disposal methods within the study areas.

		Pick up by garbage truck	Open dumping	Burning	Total
Community	Buipela	37 (33.9%)	51 (46.8%)	21 (19.3%)	109 (100%)
	Fuo	12 (41.4%)	14 (48.3%)	3 (10.3%)	29 (100%)
	Kanvili	50 (37.9%)	59 (44.7%)	23 (17.4%)	132 (100%)
Total		99 (36.7%)	124 (45.9%)	47 (17.4%)	270 (100%)

## Data Availability

The data used to support the findings of this study are available from the corresponding author upon request.
